# MC-ViT: Multi-path cross-scale vision transformer for thymoma histopathology whole slide image typing

**DOI:** 10.3389/fonc.2022.925903

**Published:** 2022-10-31

**Authors:** Huaqi Zhang, Huang Chen, Jin Qin, Bei Wang, Guolin Ma, Pengyu Wang, Dingrong Zhong, Jie Liu

**Affiliations:** ^1^ School of Computer and Information Technology, Beijing Jiaotong University, Beijing, China; ^2^ Department of Pathology, China-Japan Friendship Hospital, Beijing, China; ^3^ Department of Radiology, China-Japan Friendship Hospital, Beijing, China; ^4^ School of Information Science and Engineering, East China University of Science and Technology, Shanghai, China

**Keywords:** thymoma typing, histopathology whole slide image, vision transformer, cross-correlation attention, multi-scale feature fusion

## Abstract

**Objectives:**

Accurate histological typing plays an important role in diagnosing thymoma or thymic carcinoma (TC) and predicting the corresponding prognosis. In this paper, we develop and validate a deep learning-based thymoma typing method for hematoxylin & eosin (H&E)-stained whole slide images (WSIs), which provides useful histopathology information from patients to assist doctors for better diagnosing thymoma or TC.

**Methods:**

We propose a multi-path cross-scale vision transformer (MC-ViT), which first uses the cross attentive scale-aware transformer (CAST) to classify the pathological information related to thymoma, and then uses such pathological information priors to assist the WSIs transformer (WT) for thymoma typing. To make full use of the multi-scale (10×, 20×, and 40×) information inherent in a WSI, CAST not only employs parallel multi-path to capture different receptive field features from multi-scale WSI inputs, but also introduces the cross-correlation attention module (CAM) to aggregate multi-scale features to achieve cross-scale spatial information complementarity. After that, WT can effectively convert full-scale WSIs into 1D feature matrices with pathological information labels to improve the efficiency and accuracy of thymoma typing.

**Results:**

We construct a large-scale thymoma histopathology WSI (THW) dataset and annotate corresponding pathological information and thymoma typing labels. The proposed MC-ViT achieves the Top-1 accuracy of 0.939 and 0.951 in pathological information classification and thymoma typing, respectively. Moreover, the quantitative and statistical experiments on the THW dataset also demonstrate that our pipeline performs favorably against the existing classical convolutional neural networks, vision transformers, and deep learning-based medical image classification methods.

**Conclusion:**

This paper demonstrates that comprehensively utilizing the pathological information contained in multi-scale WSIs is feasible for thymoma typing and achieves clinically acceptable performance. Specifically, the proposed MC-ViT can well predict pathological information classes as well as thymoma types, which show the application potential to the diagnosis of thymoma and TC and may assist doctors in improving diagnosis efficiency and accuracy.

## Introduction

Thymic epithelial tumors (i.e., thymomas) are uncommon and primary anterior mediastinum neoplasms derived from the thymic epithelium. According to the histological classification standard, the World Health Organization (WHO) distinguishes thymomas (types A, AB, B1, B1+B2, B2, B2+B3, and B3) from thymic carcinoma (TC) (1, 2). Considering that thymoma may gradually develop into TC, thymoma typing is crucial to assist doctors in diagnosis and prognosis (3). The morphological diagnosis of thymoma has traditionally posed difficulties for histopathologists since thymoma has great histological variability and intratumoral heterogeneity (4, 5), and it is difficult to conceptualize a cogent and easily reproducible morphological classification standard. Currently, based on the schema of WHO, the morphological classification of thymic epithelial neoplasms is described as follows: Type A thymoma usually consists of the spindle or ovoid-shaped cells with bland nuclei, scattered chromatin, and inconspicuous nucleoli arranged in solid sheets with few or no lymphocytes in the tumor. By comparison, type B thymoma may display coarse lobulation delineated by fibrous septa. Type B1 thymoma contains dense lymphocyte neoplastic with scant neoplastic epithelial cells, which are composed of oval cells with pale round nuclei and small nucleoli. In type B2 thymoma, the neoplastic thymic epithelial cells are increased in number and appear as scattered plump cells among equivalent mixed lymphocytes. The epithelial cells are large and polygonal, which have obvious vesicular nuclei and central prominent nucleoli, and show a tendency to palisade around vessels and fibrous septa. Here, dilated perivascular spaces are commonly existed. Type B3 thymoma corresponds to the lobular growth pattern of a smoothly contoured tumor composed predominantly of epithelial cells having a round or polygonal shape and clear cytoplasm. Note that perivascular spaces with epithelial palisading are prominent, and lymphocytes are almost always interspersed among the tumor cells. In addition, type AB thymoma has features of type A thymoma that are admixed with foci showing features of type B thymoma. TC exhibits clear-cut cytological atypia and a set of cytoarchitectural features no longer specific to the thymus (6).

At present, the diagnosis of thymoma and TC basically relies on the visual observation of WSIs by histopathologists. With the rapid development of deep learning technology, we aim to develop a computer-assisted diagnosis (CAD) system to provide doctors with more histopathological information to assist the diagnosis and prognosis. More specifically, we can achieve the initial screening of WSIs through an efficient CAD system (7–10) to assist doctors in obtaining the detailed thymoma pathological information and the accurate thymoma typing results. Over the past few years, convolutional neural networks (CNNs) have shown excellent performance in most computer vision tasks including medical image processing. However, many studies (11–13) have gradually discovered some inherent limitations of CNNs, such as the difficulty in modeling long-range dependencies and the local receptive field. To better modeling global feature relations, some scholars extend the transformer from the natural language processing field to the computer vision field, and then propose high-performance vision transformers (ViTs) including Swin-T (12), PVT (13), LeViT (14), TNT (15), T2T-ViT (16), IPT (17), and Uformer (18) to serve various high-level and low-level vision tasks. In addition, there are also some ViT variants developed to achieve medical image processing, such as GasHis-ViT (19) for histopathology image normal and abnormal classification, and Swin-Unet (20) and AFTer-Unet (21) for multi-organ CT image segmentation. However, in digital pathology workflow, existing ViTs are difficult to effectively utilize for thymoma histopathology WSI typing due to the following two problems (1): Affected by the implementation mechanism of multi-head self-attention (MSA), current ViTs usually have large computational costs; thus, it is unsuitable to directly process the full-scale WSI with millions of resolutions (2). Although many existing ViTs can effectively model global and local feature relations, most of them fail to employ the complementary between multi-scale or multi-resolution features. Considering that thymoma histopathology WSIs have the inherent multi-scale information, for example, a WSI includes three magnification versions in terms of 10 × , 20 × , and 40 × . Moreover, the local pathological information of a WSI has close correspondences with the thymoma type. Therefore, we can address such problems by comprehensively employing the above-mentioned two types of information to design ViT.

In this paper, we propose a multi-path cross-scale vision transformer (MC-ViT) to achieve thymoma histopathology WSI typing. MC-ViT contains two core components, the first one named cross attentive scale-aware transformer (CAST), which takes the multi-scale patches from the same WSI as inputs and then predicts corresponding pathological information classes (spindle thymic epithelial cells, B1 thymic epithelial cells, B2 thymic epithelial cells, B3 thymic epithelial cells, fibrous septa, erythrocyte, lymphocyte, perivascular space, medullary differentiated areas, and tumor) to serve thymoma typing. Unlike the standard ViT (11), the proposed CAST constructs multiple paths to separately process 10 × , 20 ×, and 40 × WSI patches for capturing potential pathological information in different receptive field features. In general, 10 × WSI patches contain more information about the medullary differentiated areas and fibrous septa, 20 × WSI patches are mainly related to the perivascular space and lymphocyte, and 40 × WSI patches can better reflect the properties of the erythrocyte and thymic epithelial cells. To comprehensively utilize such pathological information, we also propose a cross-correlation attention module (CAM) to fuse multi-scale features in the main path of CAST. The second component is the WSIs transformer (WT), which is designed to classify the thymoma type of WSIs. Here, we propose to use the fixed number of multi-scale WSI patches to represent a full-scale WSI, and introduce the pathological information labels of these WSI patches as priors to improve the interpretability and accuracy of thymoma typing. Specifically, we concatenate the low-level features of multi-scale WSI patches and corresponding pathological information labels to form a 1D feature matrix as the input, and then predict the thymoma type (A, AB, B1, B1+B2, B2, B2+B3, B3, or C) by WT. Based on this design, we achieve 95.1% thymoma typing accuracy using a lightweight model with only a three-stage transformer encoder. Finally, this paper constructs a large-scale thymoma histopathology WSI (THW) dataset, which contains 129 hematoxylin & eosin (H&E)-stained WSIs with the pathological information and thymoma typing annotations.

**Figure 1:**
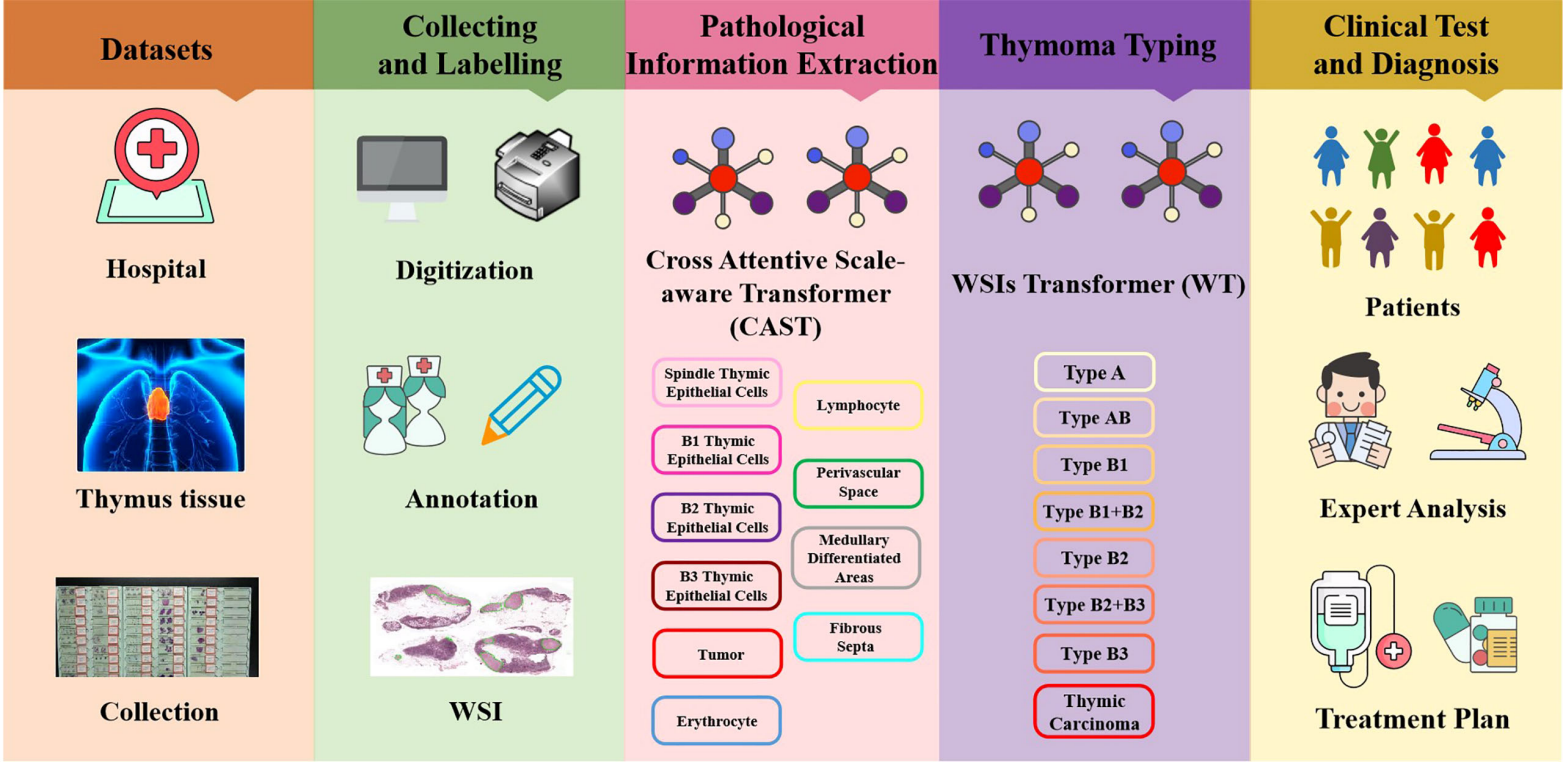
Illustration of the thymoma diagnosis workflow. Firstly, we collect the clinical data from the China–Japan Friendship Hospital to construct the thymoma histopathology WSI (THW) dataset. Then, histopathologists are invited to manually label the WSIs of the THW dataset as eight thymoma types with 10 classes of local pathological information. Next, we propose the cross attentive scale-aware transformer (CAST) for pathological information classification, which can guide the WSI transformer (WT) to achieve accurate thymoma histopathology WSI typing. Finally, according to the predicted results of the network, doctors can more efficiently and accurately diagnose thymoma and TC.

The thymoma diagnosis workflow is illustrated in **Figure 1**, and the main contributions can be summarized as follows:

- We propose an MC-ViT, which is the first transformer architecture designed for thymoma histopathology WSI typing.
- We develop a CAST with a cross-correlation attention mechanism, which can fully leverage the multi-scale information inherent in WSIs to achieve pathological information classification.
- We achieve the end-to-end thymoma histopathology WSI typing. The proposed WSIs transformer takes pathological information labels as priors to convert a WSI into a 1D feature matrix as the network input, which solves the computing complexity problem caused by full-scale WSI.
- We publish a large-scale thymoma histopathology WSI dataset with 323 H&E-stained WSIs from 129 patients and annotate the pathological information classes and thymoma types.

## Related works

### Vision transformers

Starting with AlexNet (22), deep CNNs serve as the mainstream backbone networks in computer vision for many years. However, many studies (11–13) point out that CNNs are unsuitable to model long-range dependencies in the data. Recently, with the development of the non-local self-attention, the transformer (23) and its variants (12–15, 17, 18) show excellent performance on many computer vision tasks and the potential to replace CNNs. For example, ViT (11) adopts the classical transformer architecture [23] to achieve image classification; it first splits an image into non-overlapping patches and then regards these patches as input tokens for network training. To reduce the model complexity of the vision transformer, Swin-T (12) proposes an efficient shifted-window-based self-attention, and adopts two successive Swin transformer blocks to model non-local feature relations. For achieving dense prediction tasks (e.g, instance segmentation and object detection), Wang et al. (13) design the Pyramid Vision Transformer (PVT) and the Spatial-Reduction Attention (SRA) to effectively reduce resource consumption and computational costs of using transformer. Moreover, in high-level vision tasks, LeViT (14) develops an alternately residual block and employs the attention bias to replace traditional absolute position embeddings for achieving competitive performance. After that, transformer in transformer (TNT) (15) combines the patch-level and pixel-level transformer blocks; thus, this architecture can effectively represent the feature relations between and within regions. In low-level vision tasks, Chen et al. (17) not only construct a large-scale benchmark based on the ImageNet dataset, but also design an image processing transformer (IPT) to serve various image restoration tasks including image super-resolution, denoising, and deraining. Then, Uformer (18) presents a hierarchical U-shaped transformer architecture with skip connections like U-Net (24). By combining the depth-wise convolution in basic transformer blocks, Uformer can capture long-range and short-range dependencies (global and local information) simultaneously. However, the above vision transformers fail to comprehensively consider the multi-scale information of an image. In this paper, we further propose an MC-ViT, which can effectively extract and employ multi-scale features to improve network performance.

### Attention mechanism

In deep learning-based methods, the attention mechanism can enhance important features as well as suppress redundant features, thereby improving the network performance on various computer vision tasks. In general, attention mechanisms are mainly divided into three classes according to different modes of action (1): channel attention, (2) spatial attention, and (3) self-attention. In addition to the self-attention mechanism mentioned above, it is worth noting that the Squeeze-and-Excitation (SE) module (25) is the first plug-and-play channel attention mechanism, which can model the cross-channel interdependence to enhance the useful channels of features. Motivated by the SE module, selective kernel network (SKNet) (26) presents to use the multi-scale information with different receptive fields to adjust the weights of the channel attention. Subsequently, Woo et al. (27) design a convolutional block attention module, which not only proposes spatial attention to enhance important feature locations by aggregating neighborhood information, but also combines spatial attention and the channel attention for achieving attention complementarity. The similar spatial attention is also used in the Attention-UNet (28). In addition, triplet attention (29) and tensor element self-attention (30) can establish the cross-dimension feature interactions for achieving multi-view spatial attention. More recently, to model the attention across multi-scale features, cross-MPI (31) presents to use the batch-wise multiplication to explicitly correlate input features and corresponding multi-depth planes. Different from the above methods, we develop an efficient cross-correlation attention module in CAST; this attention mechanism can model the spatial-level multi-scale feature relations and then enhance the multi-scale fusion features at each transformer block. Extensive experiments also demonstrate that the proposed CAM is effective to improve the network performance on the thymoma typing task.

## Materials and methods

### Patients and dataset

**Figure 2:**
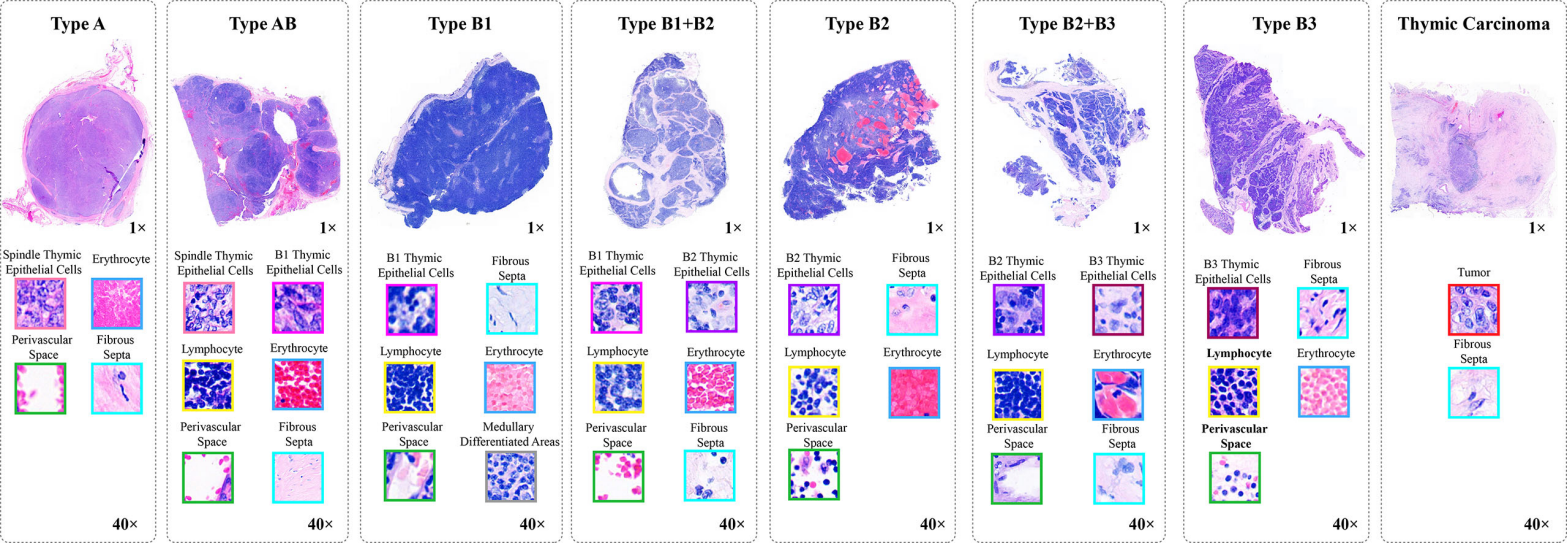
The pathological information against the thymoma types. Concretely, there are 10 pathological information classes (spindle thymic epithelial cells, B1 thymic epithelial cells, B2 thymic epithelial cells, B3 thymic epithelial cells, fibrous septa, erythrocyte, lymphocyte, perivascular space, medullary differentiated areas, and tumor) and eight types (A, AB, B1, B1+B2, B2, B2+B3, B3 and TC).

In this study, all content, including the informed consent of patients, received approval from the Institutional Ethics Review Committee of the China–Japan Friendship Hospital. Specifically, we collected 323 H&E-stained whole slides from 129 thymoma and TC patients, and show the clinical information of such patients in **Table 1**. Afterwards, we produced corresponding thymoma histopathology WSIs by scanning these slides through the high-throughput digital scanner Shenzhen Shengqiang Technol. Co. Ltd (Slide Scan SystemSQS-600P). Each WSI has three magnification scales in terms of 10×, 20× and 40× with resolutions of 0.57 μm/pixel , 0.29 μm/pixel , and 0.14 μm/pixel , respectively. To obtain accurate thymoma typing annotations, we invited experienced pathologists to label such WSIs as eight thymoma types, as shown in **Figure 2**, namely, type A, type AB, type B1, type B1+B2, type B2, type B2+B3, type B3, and TC. Considering the morphological continuum characteristic of thymomas, it remains a challenge to effectively distinguish the types B1, B2, and B3 thymomas. At present, the manual annotation of thymomas is mainly dependent on the experience and subjective judgment of pathologists, so there is usually a certain difference between the annotation results of different pathologists. To improve the annotation quality of the training set, the invited pathologists use the collective discussion to determine the type of each patient, and during the annotation, they check the corresponding immunohistochemistry (IHC)-stained WSI of each H&E-stained WSI to define a more accurate thymoma type. In addition, different pathological information related to thymoma typing is also labeled on WSIs, including spindle thymic epithelial cells, B1 thymic epithelial cells, B2 thymic epithelial cells, B3 thymic epithelial cells, fibrous septa, erythrocyte, lymphocyte, perivascular space, medullary differentiated areas, and tumor. For some indistinguishable classes like thymic epithelial cells (B1, B2, and B3), we provide the corresponding IHC-stained WSIs, which can help us locate thymic epithelial cells and calculate the ratio between epithelial cells and lymphocytes in local WSI regions. Concretely, the number of lymphocytes is more than that of epithelial cells in B1 thymoma WSIs, the number of lymphocytes is close to that of epithelial cells in B2 thymoma WSIs, and the number of lymphocytes is lower than that of epithelial cells in B3 thymoma WSIs. Furthermore, there are still slight differences in the nuclear heterogeneity, cell size, and chromatin for thymic epithelial cells (B1, B2, and B3). The above properties can also assist pathologists in distinguishing the thymoma type of a WSI. In this way, we consider the epithelial cells in B1, B2, or B3 thymoma WSIs as B1, B2, or B3 thymic epithelial cells. As shown in **Figure 2**, a total of 10 classes of pathological information can be used to train the proposed MC-ViT for improving the accuracy of thymoma typing. After that, we denote these labeled data as the thymoma histopathology WSI dataset, where 243 WSIs are selected to train the proposed pipeline and 80 other WSIs are used as the test set. Among them, each WSI is divided into 3,000 non-overlapping patches with three resolutions (64×64, 128×128, and 256×256) for network training. By constructing this large-scale dataset, we can effectively achieve the thymoma histopathology WSI typing to further assist doctors in diagnosing thymoma or TC.

**Table 1:** Clinical information of patients.

	Basic information of patients		Thymoma typing information of patients							
Male	Female	Age	A	AB	B1	B1+B2	B2	B2+B3	B3	TC
61	68	17–81	12	30	15	18	20	9	19	6

### Overall architecture

**Figure 3:**
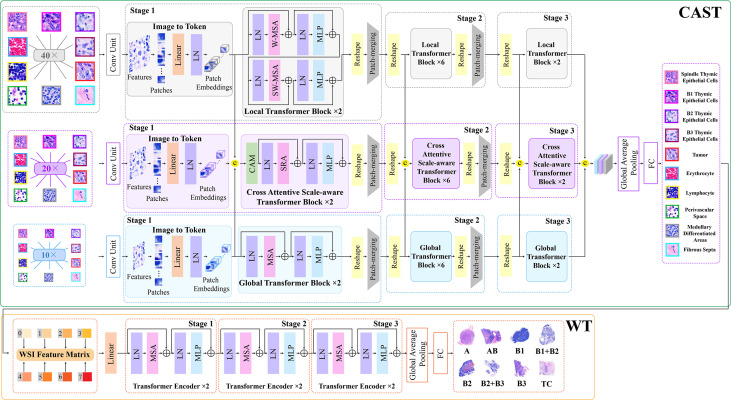
The architecture of the proposed multi-path cross-scale vision transformer (MC-ViT), which consists of the cross attentive scale-aware transformer (CAST) for pathological information classification and the WSIs transformer (WT) for thymoma typing.

Thymoma typing is a complex and challenging digital pathology workflow. As shown in **Figure 2**, doctors usually need to comprehensively consider different local pathological information from multi-scale (10×, 20×, and 40× magnifications) WSIs to confirm the thymoma type. Therefore, taking local pathological information as priors can effectively achieve the deep learning-based thymoma histopathology WSI typing. In this paper, we propose an MC-ViT and show its overall architecture in **Figure 3**. Concretely, the proposed MC-ViT consists of two sub-networks: (1) the CAST for pathological information classification, and (2) the WSIs transformer for thymoma typing.

**Figure 4:**
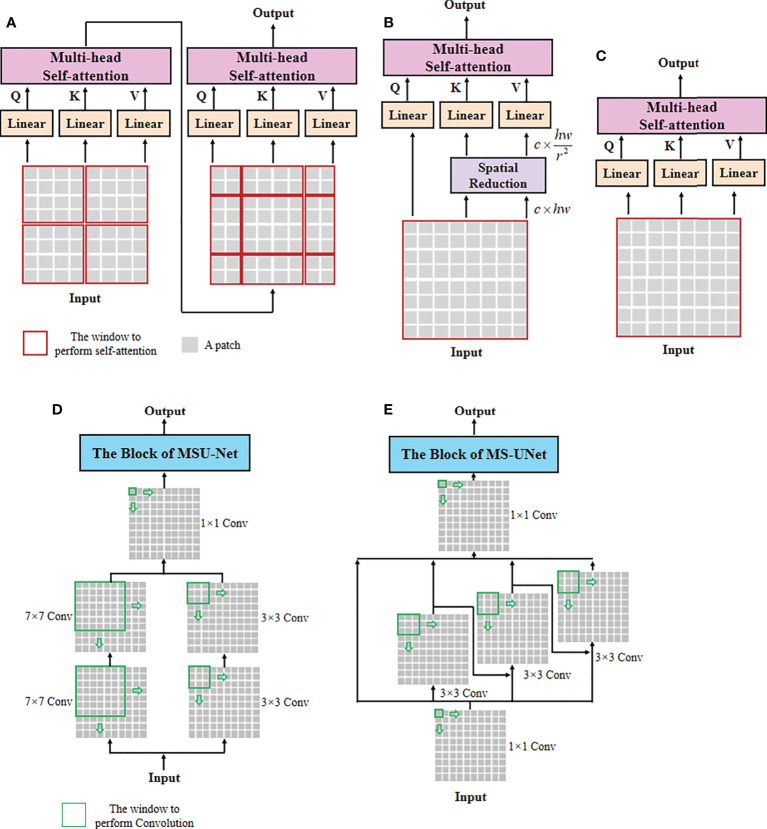
The architectures of self-attention and multi-scale convolution. **(A)** Window-based multi-head self-attention (W-MSA), **(B)** spatial reduction attention (SRA), **(C)** multi-head self-attention (MSA), **(D)** multi-scale convolution of MSU-Net, and **(E)** multi-scale convolution of MS-UNet.

In the concrete implementation, each WSI is firstly split into 10×, 20× and 40× magnification WSI patches with sizes of *H*/2×*W*/2×3 , *H*×*W*×3, and 2*H*×2*W*×3 , respectively, where multi-scale WSI patches at the same position on a WSI can form a group of network inputs, and H and W represent the height and width of WSI patches. The first sub-network CAST is designed as a three-branch structure, where the local-guided branch (LGB) and the global-guided branch (GGB) can extract the local and global receptive field features from 40× and 10× WSI patches, respectively, and the feature aggregation branch (FAB) takes 20× WSI patches as inputs. In the above branches, we first use a patch splitting layer to split and flatten input WSI patches into non-overlapping 1D features, and then adopt a linear embedding layer to project these 1D features to the expected dimensions, like Swin-T (12), where each group of 1D features can be regarded as a “token”. After that, we utilize three well-established transformer architectures including Swin-T (12), PVT (13), and ViT (11) to construct LGB, FAB, and GGB, respectively, for adapting multi-scale input features. Concretely, each branch is built as a hierarchical structure with three stages, LGB, FAB, and GGB, which respectively use the window-based multi-head self-attention (W-MSA), the spatial reduction attention (SRA), and the multi-head self-attention (MSA) to build basic transformer blocks as shown in **Figure 4**, and adopt the patch splitting layer with 4×4 kernel size to achieve two times down-sampling for token sequences to produce hierarchical representations. The configurations of each network branch are illustrated in **Table 2**. Different from LGB and GGB, to effectively predict pathological information classes of input WSI patches, the FAB fuses multi-scale (multiple receptive fields) features from different branches at each transformer block. Here, we carefully design a cross-correlation attention module, which can establish the spatial-level relations between multi-scale features with potential pathological information, thereby promoting the multi-scale feature fusion in the transformer.

**Table 2:** The network configurations of the proposed MC-ViT, where *P*, *C*, *N*, and *E* indicate the patch size, the channel number of the output, the head number of transformer block, and the expansion ratio of MLP, respectively.

		Stage	Branch	Input size	Patch merging	Transformer encoder	Output size
MC-ViT	CAST	Stage 1	LGB (40×)	256^2^×3	*P* = 8, *C* = 128	$\left[\begin{array}{c} N=2\\ E=8 \end{array}\right]\times 2$	32^2^×128
			FAB (20 ×)	128^2^×3	*P* = 4, *C* = 128	$\left[\begin{array}{c} N=2\\ E=8 \end{array}\right]\times 2$	32^2^×128
			GGB (10 ×)	64^2^×3	*P* = 2, *C* = 128	$\left[\begin{array}{c} N=2\\ E=8 \end{array}\right]\times 2$	32^2^×128
		Stage 2	LGB (40 ×)	32^2^×128	*P* = 2, *C* = 256	$\left[\begin{array}{c} N=4\\ E=4 \end{array}\right]\times 2$	16^2^×256
			FAB (20 ×)	32^2^×128	*P* = 2, *C* = 256	$\left[\begin{array}{c} N=4\\ E=4 \end{array}\right]\times 2$	16^2^×256
			GGB (10 ×)	32^2^×128	*P* = 2, *C* = 256	$\left[\begin{array}{c} N=4\\ E=4 \end{array}\right]\times 2$	16^2^×256
		Stage 3	LGB (40 ×)	16^2^×256	*P* = 2, *C* = 512	$\left[\begin{array}{c} N=8\\ E=4 \end{array}\right]\times 2$	8^2^×512
			FAB (20 ×)	16^2^×256	*P* = 2, *C* = 512	$\left[\begin{array}{c} N=8\\ E=4 \end{array}\right]\times 2$	8^2^×512
			GGB (10 ×)	16^2^×256	*P* = 2, *C* = 512	$\left[\begin{array}{c} N=8\\ E=4 \end{array}\right]\times 2$	8^2^×512
	WT	Stage 1	−	512×769	−	$\left[\begin{array}{c} N=12\\ E=4 \end{array}\right]\times 2$	512×769
		Stage 2	−	512×769	−	$\left[\begin{array}{c} N=12\\ E=4 \end{array}\right]\times 2$	512×769
		Stage 3	−	512×769	−	$\left[\begin{array}{c} N=12\\ E=4 \end{array}\right]\times 2$	512×769

The second sub-network WT is a simple but effective three-stage transformer encoder. For a WSI, we randomly select a fixed number of WSI patches, and then through the CAST to produce the multi-scale embeddings and the pathological information labels of these WSI patches. Specifically, we first concatenate the multi-scale embeddings of each WSI patch with the corresponding pathological information label at the channel dimension, and then connect the concatenated features of WSI patches at the node dimension. To this end, each WSI can be encoded into a feature matrix *M*∈*ℝ* ^*m*×769^ with pathological information priors to train the proposed WT, where M indicates that each WSI is divided into M small patches. In the WT, we use classical transformer blocks (11) with absolute position encodings to process the input feature matrices for thymoma typing. In addition, converting a 2D full-scale WSI to a 1D feature matrix can significantly reduce the computational costs of the transformer.

### Cross attentive scale-aware transformer

Unlike general natural and medical images (32, 33), a WSI usually has three magnification scales in terms of 10 × , 20 ×, and 40 × . To effectively utilize different scale WSIs for modeling multi-scale feature relations, we propose a CAST consisting of three kinds of basic transformer blocks, namely, the global transformer block, the CAST block, and the local transformer block. As shown in **Figure 4**, the proposed CAST is also different from existing advanced multi-scale U-Net architectures. For examples, Su et al. (34) design MSU-Net that uses scale-specific convolutions (1×1, 3×3, and 7×7) to capture multi-scale features (see **Figure 4D**). Kushnure et al. (35) construct MS-UNet to process the split feature channels to produce multi-scale representations (see **Figure 4E**). However, locally connected convolutions are not enough to extract sufficient global information, which limits the receptive fields of both MSU-Net and MS-UNet. In contrast, the proposed CAST can capture richer global information by three different non-local self-attention mechanisms, and fully leverage multi-scale WSIs (10×, 20× and 40×) rather than only the multi-scale features from a scale-specific WSI. Then, considering that the above transformer blocks have different receptive fields, the clinical observation process for thymoma histopathology WSIs can be effectively simulated in the proposed CAST. Concretely, in GGB, the global transformer block has similar configurations to that of the classical transformer block (11), which contains an MSA, a multi-layer perception (MLP), and two layer normalizations (LNs) before MSA and MLP with the GELU non-linear layers (23). The calculation process in the global transformer block is


(1) $${\tilde{A}}_{i}=\text{MSA}\left(\text{LN(}A_{i-1})\right)+A_{i-1},$$

(2) $$A_{i}=\text{MLP}\left(\text{LN(}{\tilde{A}}_{i})\right)+{\tilde{A}}_{i},$$

where *A*_*i*−1_ and *A*_*i*_ are the input and output features of the *i* th global transformer block, and ${\tilde{A}}_{i}$ denotes the output of intermediate features by the MSA.

Then, in LGB, the local transformer block continues the advantages of Swin-T (12), which replaces the MSA with the window-based multi-head self-attention, and employs two successive Swin transformer blocks to achieve cross-window connections. The concrete configurations are shown in **Figure 3**; compared with MSA, W-MSA focuses more on modeling the feature relations in non-overlapping local windows, which not only effectively promotes the extraction of local information, but also significantly reduces the computations of transformer blocks. The local transformer block can be computed as


(3) $${\tilde{B}}_{i}=\text{W}-\text{MSA}\left(\text{LN(}B_{i-1})\right)+B_{i-1},$$

(4) $${\hat{B}}_{i}=\text{MLP}\left(\text{LN(}{\tilde{B}}_{i})\right)+{\tilde{B}}_{i},$$

(5) $${\bar{B}}_{i}=\text{SW}-\text{MSA}\left(\text{LN(}{\hat{B}}_{i})\right)+{\hat{B}}_{i},$$

(6) $$B_{i}=\text{MLP}\left(\text{LN}\left({\bar{B}}_{i}\right)\right)+{\bar{B}}_{i},$$

Where *B*_*i*−1_ and *B*_*i*_ are the input and output features of the *i* th local transformer block, SW-MSA is the multi-head self-attention with the shifted windowing configuration, and ${\tilde{B}}_{i}$ , ${\hat{B}}_{i}$ , and ${\bar{B}}_{i}$ represent the intermediate features output by MSA, the first MLP, and SW-MSA, respectively. Referring to Swin-T (12), we adopt the relative position bias to compute W-MSA and SW-MSA, which can be expressed as


(7) $$\text{Self}-\text{attention(}Q,K,V)=\text{Softmax(}QK^{T}/\sqrt{d}+R)V,$$

where *Q* , *K*, and *V* are the query, key, and value matrices, respectively.

Finally, in FAB, we propose the cross-correlation attention module to combine with the spatial-reduction attention (13) to construct the cross attentive scale-aware (CAS) transformer block. Specifically, each CAS transformer block is composed of a CAM, an SRA, an MLP, and two LNs. Different from the global and local transformer blocks, we first adopt a CAM to aggregate and enhance the multi-scale features *A*_*i*_ , *B*_*i*_, and *C*_*i*_ from different branches. With this design, the spatial-level feature relations can be supplemented and the representation of potential pathological information can be boosted effectively. Then, MLP can update the multi-scale fusion features captured by SRA accompanied with LNs for stable training and rapid convergence. The CAS transformer block can be formulated as


(8) $${\tilde{C}}_{i}=\text{SRA}\left(\text{LN}\left(\text{CAM}\left(\left[A_{i-1},B_{i-1},C_{i-1}\right]\right)\right)\right)+C_{i-1},$$

(9) $$C_{i}=\text{MLP}\left(\text{LN(}{\tilde{C}}_{i})\right)+{\tilde{C}}_{i},$$

Where *C*_*i*−1_ and *C*_*i*_ are the input and output features of the *i* th CAS transformer block, and ${\tilde{C}}_{i}$ denotes the intermediate features output by the SRA.

In addition, after the last transformer block of each stage, we use a 4 × 4 patch splitting (unfolding) layer *PS*(·) to down-sample the reshaped features, and a linear embedding layer *FC*(·) to project the down-sampled features to the expected dimension for producing hierarchical representations


(10) $$A_{i}/B_{i}/C_{i}=FC\left(PS(reshape(A_{i}/B_{i}/C_{i}))\right).$$

In the proposed CAST, after fusing and updating each stage’s multi-scale features, we use the last fully connected layer with softmax of FAB to predict the pathological information classes of input WSI patches. During the test process, the predicted pathological information labels and the extracted multi-scale embeddings from the same WSI are connected as an input feature matrix to feed the subsequent WT.

### WSI transformer

Benefiting from the prediction for pathological information labels and the encoding for multi-scale embeddings by the first sub-network CAST, we can construct an efficient WT with a three-stage transformer encoder to further achieve thymoma histopathology WSI typing. As shown in **Figure 3**, after concatenating the pathological information labels and multi-scale embeddings to convert a full-scale WSI to a simple input feature matrix, the computations of WT are significantly reduced. Specifically, each stage contains two classical transformer blocks (11); the head number *N* of MSA and the expansion ratio *E* of MLP in each transformer block are set as 12 and 4, respectively. In addition, we not only introduce absolute position encodings, but also replace class tokens with a global average pooling layer and a fully connected layer (36) to improve the accuracy of thymoma typing. The network configurations of the proposed CAST and WT are shown in **Table 2**.

### Cross-correlation attention module

**Figure 5:**
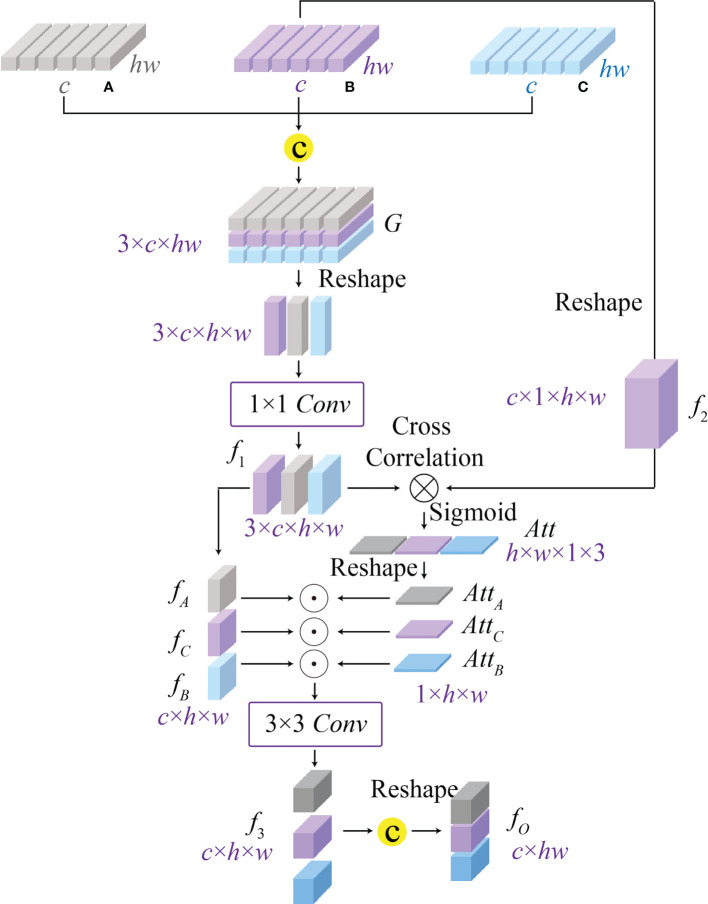
The architecture of the proposed cross-correlation attention module (CAM), which can model the spatial-level relationship between multi-scale features **(A–C)** from the global-guided, the local-guided, and the feature aggregation branches for achieving the multi-scale feature fusion.

Converting an image into a sequence of tokens will result in the spatial information loss, and most existing vision transformers (12, 13, 15, 16, 31) fail to consider the spatial-level relations between features. To address this issue, we propose a cross-correlation attention module to effectively establish the spatial-level relations between multi-scale features as well as achieving the multi-scale feature fusion. As shown in **Figure 5**, CAM can comprehensively consider different receptive field features with global and local information, and enhance the multi-scale fusion features through a spatial attention map generated by the cross-correlation attention mechanism. Considering that multi-scale features completely contain potential pathological information, the proposed CAM can further improve the accuracy of potential pathological classification. Specifically, CAM first concatenates the features *A*_*i*_∈*ℝ* ^*c*×*hw*^ , *B*_*i*_∈*ℝ* ^*c*×*hw*^ , and *C*_*i*_∈*ℝ* ^*c*×*hw*^ from GGB, LGB, and FAB, respectively, to generate the features *G*∈*ℝ* ^3×*c*×*hw*^ , and then reshapes its size to 3×*c*×*h*×*w* . After a 1 × 1 convolution, we can get the spatial-level features *f*_1_∈*ℝ* ^3×*c*×*h*×*w*^ . Moreover, we reshape the features *C*_*i*_∈*ℝ* ^*c*×*hw*^ as another spatial-level features *f*_2_∈*ℝ* ^*c*×1×*h*×*w*^ and cross-correlate features *f*_1_∈*ℝ* ^3×*c*×*h*×*w*^ and *f*_2_∈^*c*×1×*h*×*w*^ by a batch-wise multiplication to establish the relations between multi-scale features for producing the attention map *Att*


(11) $$Att=\sigma \left(conv_{1}\left(\text{reshape}\left(\left[A_{i},B_{i},C_{i}\right]\right)\right)⊗\text{reshape(}C_{i})\right),$$

Where *σ*(·) indicates the Sigmoid activation function, *conv*_1_(·) is the 1 × 1 convolution, [·, ·, ·] and reshape(·) denote the feature concatenation and reshape operations, and ⊗ is the batch-wise matrix multiplication.

After that, we split the attention map *Att*∈*ℝ* ^3×*h*×*w*^ into three individual attention maps *Att*_*A*_∈*ℝ* ^1×*h*×*w*^ , *Att*_*B*_∈*ℝ* ^1×*h*×*w*^ , and *Att*_*C*_∈*ℝ* ^1×*h*×*w*^ to enhance corresponding spatial-level features *f*_*A*_∈*ℝ* ^*c*×*h*×*w*^ , *f*_*B*_∈*ℝ* ^*c*×*h*×*w*^, and *f*_*C*_∈*ℝ* ^*c*×*h*×*w*^ by the element-wise multiplication. Here, the features *f*_*A*_ , *f*_*B*_ , and *f*_*C*_ related to different receptive field information are split from the spatial-level features *G*∈*ℝ* ^3×*c*×*h*×*w*^ . Then, we re-aggregate the enhanced spatial-level features by a 3 × 3 convolution to generate the features *f*_3_∈*ℝ* ^*c*×*h*×*w*^ . The final output *F*∈*ℝ* ^*c*×*hw*^ of CAM can be obtained by a reshape operation, and the above process is expressed as


(12) $$F=\text{reshape}\left(conv_{3}\left(\left[Att_{A}⊙f_{A},Att_{B}⊙f_{B},Att_{C}⊙f_{C}\right]\right)\right),$$

where ⊙ denotes the element-wise multiplication.

### Loss functions

The first sub-network CAST can classify input WSI patches into 10 pathological information classes, including spindle thymic epithelial cells, B1 thymic epithelial cells, B2 thymic epithelial cells, B3 thymic epithelial cells, fibrous septa, erythrocyte, lymphocyte, perivascular space, medullary differentiated areas, and tumor. Specifically, we use the cross-entropy loss (22) to train the proposed CAST, which can minimize the distance between predicted probabilities and corresponding ground truths by the following expression


(13) $${ℒ}_{\text{CAST}}=-\sum_{k=1}^{K}y_{k}\log\;(p_{k}),$$

where *K* is the number of pathological information classes, *p*_*k*_ represents the predicted probability that an input WSI patch belongs to the *k* th pathological information class, and *y*_*k*_ is its ground truth.

After that, the second sub-network WT can predict the thymoma type of the input feature matrix for achieving thymoma typing. Concretely, there are eight thymoma types (A, AB, B1, B1+B2, B2, B2+B3, B3, and C) in our task. Similarly, we also adopt the cross-entropy loss to optimize this multi-classification task as


(14) $${ℒ}_{\text{WT}}=-\sum_{t=1}^{T}Y_{t}\log\;(P_{t}),$$

where *T* is the number of thymoma types, *P*_*t*_ represents the predicted probability that a input feature matrix belongs to the *t* th thymoma type, and *Y*_*t*_ is its ground truth.

## Experimental results and analysis

### Implementation details

**Figure 6:**
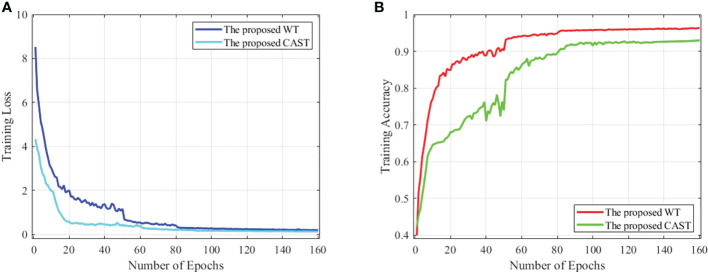
**(A)** The training loss against training epochs and **(B)** the training accuracy against training epochs on the THW dataset.

The proposed MC-ViT is programmed by PyTorch 1.9.0 and all experiments are conducted on a server with Intel (R) Core (TM) i9-10850K CPU (5.0 GHz) and NVIDIA GeForce RTX 3090 GPU (24GB). In our concrete implementation, the Adam optimizer with momentums $\bar{e}ta_{1}=0.9$ and *β*_2_=0.999 is used to optimize both CAST and WT. For the proposed CAST, there are 160 epochs in network training with batch size 64 and the initial learning rate 2*e* -3. Moreover, the proposed WT is trained in 160 epochs using batch size 8 and the initial learning rate 1*e* -3. In **Figure 6**, we report the training loss and accuracy against training epochs to show the effectiveness and convergence of the proposed CAST and WT.

### Evaluation metrics

To comprehensively evaluate the performance of the proposed CAST for pathological information classification and the performance of the proposed WT for thymus typing, we introduce eight well-established metrics, namely, recall (37) (Rec), Top-1 accuracy (Top-1 Acc), mean accuracy (38) (MAcc), precision (37) (Pre), F-measure (38) (F1), receiver operating characteristic (ROC) curve, area under the curve (AUC), and confusion matrix (CM), and three statistical metrics, namely, sensitivity and specificity with the 95% confidence interval (CI) and the two-sided McNemar’s tests (39) (test statistic and asymptotic Sig.). For the first five metrics, the larger values indicate a classification method has better performance. The AUC is defined as the area surrounded by the coordinate axis and the ROC curve, where a large AUC value denotes a high classification accuracy.

### Evaluation for pathological information classification

This subsection first compares the proposed CAST with four well-known vision transformers, ViT (11), TNT (15), LeViT (14), and CrossViT (40); two classical CNNs, ResNet-101 (41) and DenseNet-121 (42); and four state-of-the-art medical image classification methods, GuSA-Net (43), ROPsNet (44), CPWA-Net (45), and IL-MCAM (46). The quantitative results on the proposed THW dataset are shown in **Table 3**; compared with existing advanced classification methods, the proposed CAST achieves 0.016, 0.012, 0.015, and 0.007 improvements in terms of Rec, Top-1 Acc, Macc, and F1, respectively. In general, some classical transformer-based and CNN-based methods, such as ViT, TNT, LeViT, and ResNet-101, fail to achieve satisfactory classification results, which could be attributed to the fact that these methods ignore capturing and utilizing the inherent multi-scale information in WSIs. In contrast, state-of-the-art IL-MCAM and CrossViT achieve better classification accuracy since both of them are built as the multi-scale network architecture. It is noteworthy that GuSA-Net is the improvement of DenseNet; thus, its classification performance is slightly better than that of DenseNet. Currently, in most clinical cases, doctors need to comprehensively observe the multi-scale (10 × , 20 ×, and 40 × ) local patches of a WSI to determine its pathological information classes, and then diagnose the corresponding thymoma type. The proposed CAST effectively simulates the above process by taking multi-scale WSI patches as inputs and fusing multi-scale features in each stage. As a result, we successfully achieve an improvement of 0.015 on MAcc compared with the state-of-the-art IL-MCAM, and about 0.023 average improvement on other evaluation metrics.

**Table 3:** Quantitative comparisons (Rec, Top-1 Acc, MAcc, Pre, and F1) for pathological information classification on the THW dataset.

	Pathological Information Classification				
Methods	Rec	Top-1 Acc	Macc	Pre	F1
(ICLR’2021) ViT (11)	0.813	0.834	0.804	0.810	0.811
(NIPS’2021) TNT (15)	0.814	0.828	0.813	0.817	0.815
(ICCV’2021) LeViT (14)	0.821	0.857	0.833	0.827	0.824
(ICCV’2021) CrossViT (40)	0.897	0.886	0.860	0.867	0.882
(CVPR’2016) ResNet-101 (41)	0.819	0.836	0.802	0.808	0.813
(CVPR’2017) DenseNet-121 (42)	0.873	0.848	0.834	0.860	0.867
(TMI’2020) GuSA-Net (43)	0.918	0.927	0.909	0.925	0.921
(TMI’2021) ROPsNet (44)	0.874	0.892	0.886	0.882	0.878
(JBHI’2021) CPWA-Net (45)	0.817	0.832	0.821	0.813	0.815
(CBM’2022) IL-MCAM (46)	0.906	0.918	0.912	0.903	0.904
CAST (Ours)	0.934	0.939	0.927	0.922	0.928

Red and blue contents represent the best and suboptimal results, respectively.

**Figure 7:**
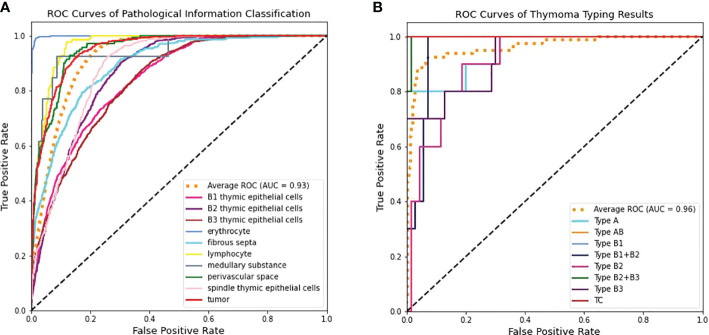
The ROC curve and AUC on the THW dataset. **(A)** Pathological information classification and **(B)** thymoma typing.

To further verify the effectiveness of the proposed CAST, we illustrate the ROC curve and AUC of each pathological information class in the left part of **Figure 7**. It can be seen that the proposed CAST performs well on six classes, namely, erythrocyte, lymphocyte, spindle thymic epithelial cells, B1 thymic epithelial cells, B2 thymic epithelial cells, and B3 thymic epithelial cells. For the other three pathological information classes, where fibrous septa and perivascular space can be distinguished on H&E-stained WSIs according to the color and position information of existing cells (like fibroblasts and erythrocytes), medullary differentiated areas are usually distinguishable on IHC-stained WSIs. Compared with the above five pathological information classes, our pipeline achieves slightly poor but still competitive classification results for fibrous septa, perivascular space, and medullary differentiated areas. In summary, the proposed CAST effectively distinguishes each pathological information class using only H&E-stained WSIs. Since the classification results of pathological information are closely related to thymoma types, CAST can assist the subsequent WT for thymoma typing.

### Thymoma typing evaluation

Clinically, the thymoma type of a WSI is reflected by multiple pathological information; hence, theoretically, the high-precision classification results for pathological information are helpful to improve the accuracy of thymoma typing. To demonstrate the above content, we respectively use the classification methods ViT, TNT, LeViT, CrossViT, ResNet-101, DenseNet-121, GuSA-Net, and IL-MCAM to predict the pathological information labels and the uniform size embeddings of each WSI. By concatenating these labels and embeddings to produce input feature matrices to train WT, we can denote corresponding comparison methods as ViT+WT, TNT+WT, LeViT+WT, CrossViT+WT, ResNet-101+WT, DenseNet-121+WT, GuSA-Net+WT, ROPsNet+WT, CPWA-Net+WT, and IL-MCAM+WT. Their predicted results are shown in **Table 4**; we can observe that the proposed MC-ViT (CAST+WT) achieves the best classification accuracy, especially on Top-1 Acc (about 0.017 improvement) and F1 (about 0.016 improvement). The ROC curve and AUC of each thymoma type are also shown in the right part of **Figure 7**, which further proves that the proposed MC-ViT is effective to classify various thymoma types. Based on the above quantitative analysis, we can conclude that the pathological information labels provided by CAST help to achieve thymoma histopathology WSI typing, and the quality of such labels and embeddings determines the typing accuracy.

**Table 4:** Quantitative comparisons (Rec, Top-1 Acc, Macc, Pre, and F1) for thymoma typing on the THW dataset.

Methods		Thymoma Typing				
	Rec	Top-1 Acc	MAcc	Pre	F1
(ICLR’2021) ViT (11)+WT	0.832	0.839	0.820	0.824	0.828
(NIPS’2021) TNT (15)+WT	0.825	0.861	0.836	0.839	0.852
(ICCV’2021) LeViT (14)+WT	0.844	0.868	0.843	0.849	0.846
(ICCV’2021) CrossViT (40)+WT	0.903	0.899	0.875	0.879	0.891
(CVPR’2016) ResNet-101 (41)+WT	0.841	0.831	0.819	0.825	0.833
(CVPR’2017) DenseNet-121 (42)+WT	0.902	0.863	0.842	0.865	0.883
(TMI’2020) GuSA-Net (43)+WT	0.931	0.937	0.916	0.934	0.923
(TMI’2021) ROPsNet (44)+WT	0.881	0.898	0.890	0.896	0.888
(JBHI’2021) CPWA-Net (45)+WT	0.848	0.856	0.843	0.846	0.847
(CBM’2022) IL-MCAM (46)+WT	0.921	0.928	0.915	0.908	0.914
CAST+WT (Ours)	0.948	0.951	0.942	0.931	0.939

Red and blue contents represent the best and suboptimal results, respectively.

In addition, we statistically analyze the performance of these comparison methods and our MC-ViT by computing the sensitivity and specificity with 95% CI, and the two-sided McNemar’s tests (test statistic and asymptotic Sig.). As can be seen from **Table 5**, the proposed MC-ViT achieves completely correct typing results (sensitivity) for types AB, B1, B2, and C, and the competitive average 0.875 sensitivity (95% CI: 0.528–0.970) and 0.982 specificity (95% CI: 0.911–0.992) for the thymoma typing task. Moreover, the two-sided McNemar’s tests (average 1.810 test statistic and 0.42996 asymptotic Sig.) further show the statistical significance of our predicted results, which have slight differences from the expert-annotated ground truths.

**Table 5:** Quantitative comparisons (sensitivity and specificity with 95% CI, test statistic, and asymptotic Sig.) for thymoma typing on the THW dataset.

Types	Sensitivity (95% CI)	Specificity (95% CI)	Test Statistic	Asymptotic Sig.
A	0.800 (0.442–0.965)	1.000 (0.935–1.000)	0.500	0.47950
AB	1.000 (0.655–1.000)	1.000 (0.935–1.000)	−	−
B1	1.000 (0.655–1.000)	0.986 (0.912–0.999)	0.000	1.00000
B1+B2	0.800 (0.442–0.965)	1.000 (0.935–1.000)	0.500	0.47950
B2	1.000 (0.655–1.000)	0.871 (0.765–0.936)	7.111	0.00766
B2+B3	0.800 (0.442–0.965)	1.000 (0.935–1.000)	0.500	0.47950
B3	0.600 (0.274–0.863)	1.000 (0.935–1.000)	2.250	0.13361
TC	1.000 (0.655–1.000)	1.000 (0.935–1.000)	−	−
Average	0.875 (0.528–0.970)	0.982 (0.911–0.992)	1.810	0.42996

## Discussion

In this section, we discuss the effectiveness of the proposed multi-scale (multi-path) transformer architecture and the cross-correlation attention mechanism. Concretely, we define eight ablation models: (1) Single-branch Swin-T Transformer (SSwT): SSwTonly has LGB for processing 40 × WSIs; (2) Single-branch Pyramid Vision Transformer (SPVT): SPVT only has FAB for processing 20 × WSIs; (3) Single-branch Vision Transformer (SViT): SViT only has GGB for processing 10 × WSIs; (4) CAST without (w/o) GGB: this model has LGB and FAB for processing 40 × and 20 × WSIs; (5) CAST w/o FAB: this model has LGB and GGB for processing 40 × and 10 × WSIs; (6) CAST w/o LGB: this model has FAB and GGB for processing 20 × and 10 × WSIs; (7) CAST w/o CAM: this model contains three paths but without CAM; and (8) the proposed CAST. For fair comparisons, the training dataset and implementation details remain unchanged, and corresponding experimental results are exhibited in the following subsections.

### Ablation study for multiple paths of transformer

Firstly, we evaluate the effectiveness of multiple paths in the proposed CAST, where LGB, FAB, and GGB are ablated and adopted respectively to demonstrate their contributions. The quantitative results of seven ablation models, SSwT, SPVT, SViT, CAST w/o GGB, CAST w/o FAB, CAST w/o LGB, and CAST, are listed in **Table 6**. It can be seen that ablating GGB reduces the accuracy to classify the medullary differentiated areas and fibrous septa, ablating LGB weakens the performance to distinguish different thymic epithelial cells, and ablating FAB causes unsatisfactory results to recognize the perivascular space and lymphocyte. In summary, simultaneously adopting three paths in CAST to process multi-scale WSIs can achieve more excellent performance compared with using a single path or dual paths, and FAB brings the largest improvement to pathological information classification.

**Table 6:** Ablation study (Acc and MAcc) for multiple paths in the proposed CAST on the THW dataset.

Acc of Each Class	SSwT	SPVT	SViT	CAST w/o GGB	CASTw/o FAB	CASTw/o LGB	CAST
Spindle Thymic Epithelial Cells	0.859	0.871	0.844	0.866	0.918	0.897	0.923
B1 Thymic Epithelial Cells	0.887	0.876	0.854	0.895	0.886	0.905	0.915
B2 Thymic Epithelial Cells	0.893	0.881	0.861	0.903	0.892	0.911	0.920
B3 Thymic Epithelial Cells	0.890	0.879	0.858	0.898	0.887	0.909	0.916
Fibrous Septa	0.859	0.877	0.896	0.915	0.919	0.902	0.924
Erythrocyte	0.912	0.918	0.904	0.925	0.926	0.933	0.941
Lymphocyte	0.861	0.896	0.849	0.902	0.924	0.927	0.938
Perivascular Space	0.865	0.894	0.842	0.898	0.917	0.929	0.936
Medullary Differentiated Areas	0.857	0.883	0.905	0.918	0.921	0.905	0.927
Tumor	0.887	0.886	0.882	0.897	0.905	0.908	0.929
MAcc	0.877	0.886	0.870	0.902	0.910	0.913	0.927

### Ablation study for multi-scale transformer architecture

**Figure 8:**
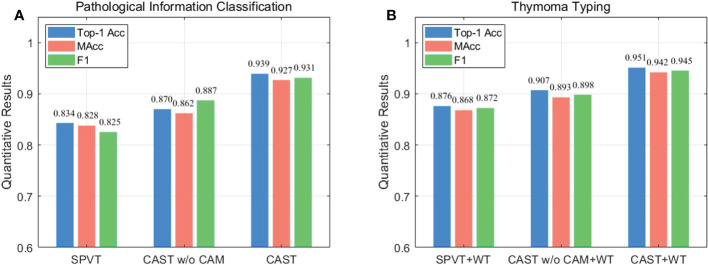
Ablation study (Top-1 Acc, MAcc, and F1) on the THW dataset, where w/o represents without such component. **(A)** Pathological information classification and **(B)** thymoma typing.

**Figure 9:**
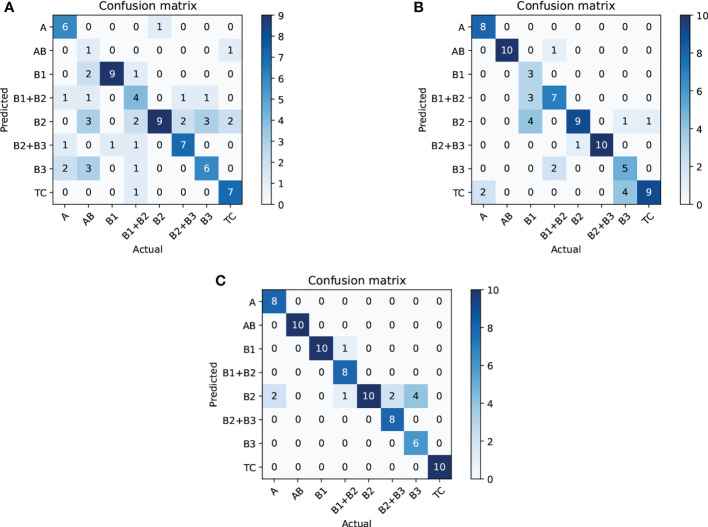
Ablation study (confusion matrix) for thymoma typing on the THW dataset; subfigures **(A–C)** are SPVT+WT, CAST w/o CAM+WT, and CAST+WT, where w/o represents without such component.

Next, we compare CAST w/o CAM and SPVT to verify the effectiveness of the proposed multi-scale transformer architecture. Specifically, CAST w/o CAM adopts three transformer branches to process 10×, 20× and 40× WSI patches, respectively, while replacing the proposed CAM by the traditional feature concatenation to achieve multi-scale feature fusion. SPVT only uses a single branch with the SRA-based transformer blocks to train 20 × WSI patches. The quantitative comparisons (Top-1Acc, Macc, and F1) on the THW dataset are reported in **Figure 8A**, and it can be seen that using the multi-scale transformer architecture brings significant performance improvements for pathological information classification. In addition, **Figure 9** shows their confusion matrices, which further demonstrate that comprehensively considering the multi-scale information in WSIs can reduce the confusion between similar thymoma types.

### Ablation study for cross-correlation attention mechanism

Finally, we evaluate the contribution of the proposed CAM to show its effectiveness on pathological information classification and thymoma typing. Corresponding quantitative results are shown in **Figure 8**, and from the overall integration of evaluation metrics Top-1 Acc, Macc, and F1, we can observe that adopting CAM to aggregate multi-scale features significantly improves the precision for pathological information classification and thymus typing. On the other hand, the confusion matrices about thymoma typing are reported in **Figure 9**; although CAST w/o CAM+WT outperforms SPVT+WT, some highly similar thymoma types are still difficult to distinguish, such as B1, B1+B2, and B2 types. By comparison, the proposed MC-ViT (CAST+WT) achieves better thymoma typing results. Overall, these ablation studies show that the accurate pathological information labels are beneficial for boosting thymoma typing accuracy, and the proposed CAM is effective to improve pathological information classification results.

### Unsupervised method for thymoma typing

In CAD, unsupervised methods are mainly used for processing unlabeled or incompletely labeled data, and they can automatically determine the total class of input data and then achieve the classification task. Traditional unsupervised methods include clustering and dimensionality reduction, and deep learning-based unsupervised methods include domain adaptation and contrastive learning. Compared with traditional methods, most deep learning-based methods have superior performance but require minor annotation information to assist network training, which means they fail to achieve full unsupervised classification. For example, domain adaptation methods require a small labeled dataset as the source domain to achieve the unsupervised classification of the target domain. Contrastive learning methods need to define the similarity between samples through pretext tasks, and then classify these data in a self-supervised (unsupervised) way. In general, supervised methods perform favorably against full unsupervised methods. In this work, we introduce a classical full unsupervised method (47) for thymoma typing, which is the combination of CNN and *k* -means clustering. We find that this method cannot successfully distinguish types B1, B1+B2, B2, B2+B3, and B3; however, it still shows high potential when only classifying three types A, B, and TC (0.659 Top-1 Acc). Hence, we think that full unsupervised methods are more suitable for a simple classification of unlabeled data; they can provide certain diagnosis information for doctors while effectively reducing the time consumed by manual annotation. By comparison, supervised methods can better achieve precise thymoma typing when having sufficient labeled data.

## Conclusions

In this paper, we propose an MC-ViT for achieving thymoma histopathology WSI typing. Aiming at full-scale WSIs that are difficult to train by deep learning-based methods, the proposed MC-ViT is designed as a twofold transformer architecture to separately predict the pathological information labels of WSI patches and the thymoma type of a WSI, where the former effectively fuses complementary multi-scale information to produce accurate pathological information priors, and the latter successfully converts the full-scale WSI to the low-cost feature matrix to achieve efficient network training by introducing such priors. In addition, we propose a cross-correlation attention mechanism to enhance and fuse multi-scale features with global and local receptive fields. Considering that CAM well establishes the spatial-level feature relations in the transformer, our thymoma typing results achieve further improvements. Extensive experiments also show that our MC-ViT outperforms most existing advanced transformer-based and CNN-based methods on the proposed THW dataset with 323 WSIs. In future works, we look forward to incorporating CT images and histopathology WSIs for achieving the multi-modal information fusion-based thymoma typing, which may further assist doctors to improve the efficiency and accuracy of thymoma and TC diagnosis. In addition, we will make the network outputs the soft labels (the probability of a WSI belongs to types B1, B2, and B3) instead of the hard labels (the class of a WSI belongs to type B1, B2, or B3) for thymoma WSIs with B1, B2, and B3 types, thereby providing more reasonable diagnosis information for doctors.

## Data availability statement

The datasets presented in this study can be found in online repositories. The names of the repository/repositories and accession number(s) can be found in the article/supplementary material.

## Ethics statement

This study was approved by the institutional ethics review committee of the China–Japan Friendship Hospital. Written informed consent to participate in this study was provided by the participant’s legal guardian.

## Author contributions

HZ proposed the algorithm and wrote the manuscript. HC provided the guidance and labeled the pathological information classes and thymoma types of WSIs. JQ collected the dataset from the China–Japan Friendship Hospital. BW, GM, and DZ verified the medical research significance of this study. PW designed the figures and experiments, and revised the manuscript. JL provided the financial support and guided the study. All authors contributed to the article and approved the submitted version.

## Funding

This study was supported by the National Key Research and Development Program of China (No. 2017YFA0700401), the National Natural Science Foundation of China (No. KKA309004533, 81571836), and the Fundamental Research Funds for the Central Universities (2021YJS036).

## Conflict of interest

The authors declare that the research was conducted in the absence of any commercial or financial relationships that could be construed as a potential conflict of interest.

## Publisher’s note

All claims expressed in this article are solely those of the authors and do not necessarily represent those of their affiliated organizations, or those of the publisher, the editors and the reviewers. Any product that may be evaluated in this article, or claim that may be made by its manufacturer, is not guaranteed or endorsed by the publisher.
